# Hahnemann’s Three Precautions

**Published:** 1885-05

**Authors:** C. W. Boyce

**Affiliations:** Auburn, N. Y.


### HAHNEMANN'S THREE PRECAUTIONS.

C. W. Boyce, M. D., Auburn, N. Y.

Hahnemann's *Organon* and his *Chronic Diseases* are in their teachings interchangeable. The fifth German edition of the *Organon* and the second German edition of the *Chronic Diseases* are our authority for what we know of Homœopathy—at least as Hahnemann taught it. Two editions of the *Organon*, the fourth and fifth, have been translated into English. The second edition of the *Chronic Diseases* has been translated into English. In order of time the first four editions of the *Organon* came out. Following these in 1828 the first volume of the first edition of the *Chronic Diseases* was published. This contained four volumes. The last appeared in 1830. In the following three years the fifth edition of the *Organon* was prepared, and in 1833 Hahnemann wrote the preface and it was finished. With no loss of time he went on preparing a second edition of *Chronic Diseases*, the first volume of which appeared in 1835. This edition contained five volumes; the fifth came out in 1839. Subsequently he prepared a sixth edition of the *Organon* which has never been published. In 1865 Dr. Lutze published what he called a sixth edition. This never went into circulation, owing to a vigorous protest by the homœopathic physicians of the world, in which Madame Hahnemann joined. She wrote to the *Allegemeine Hom. Zeitung*, saying: "The time has come to publish the genuine and true *Organon*, and I will give it to the press." In the same letter she wrote: "No one, save myself, has the right to publish the sixth edition of the *Organon;* I alone possess the manuscript of this important work written by my husband's own hand; to me alone and exclusively were confided the improvements which the author made in the *Organon*." This edition has not appeared. These data show that these important works were in the process of development, step by step, during several of the best years of Hahnemann's life. First, the four editions of the *Organon;* second, the first edition of *Chronic Diseases;* third, the fifth edition of the *Organon;* fourth, the second edition of *Chronic Diseases*, and fifth, the sixth edition of the *Organon*. Dunham made an earnest effort to gain possession of this edition but failed. The second edition of the *Chronic Diseases*, as far as Hahnemann's instruction goes, is final, for this is the last work of his published.

In the first edition of *Chronic Diseases* Hahnemann cautions physicians against making what he calls two mistakes, viz.: not to give too large a dose and not to repeat too soon. In the second edition he adds a third caution. These three precautions are the subject of our discourse to-day.

On page 152, in the second paragraph of the English edition of *Chronic Diseases*, these mistakes are portrayed. "There are three mistakes which the physician cannot too carefully avoid; the first is to suppose that the doses which I have indicated as the proper doses in the treatment of chronic diseases, and which long experience and close observation have induced me to adopt, are too small; the second great mistake is the improper use of a remedy; and the third mistake consists in not letting the remedy act a sufficient length of time." We must remember that to-day we are to discuss what Hahnemann taught first, last, and all the time, and not what the chairman or secretary or any other member practices. This will account for the many extracts made from Hahnemann's writings in this article.

In section 3 of the *Organon*, Hahnemann displays the foundation upon which the entire superstructure of his art of healing is built. "The physician should distinctly understand the following conditions: What is curable in diseases in general and in each individual case in particular; that is the recognition of disease. He should clearly comprehend what is curative in drugs in general and in each drug in particular; that is, he should possess a perfect knowledge of medicinal powers. He should be governed by distinct reasons, in order to insure recovery, by adapting what is curative in medicines to what he has recognized as undoubtedly morbid in a patient; that is to say, he should adapt it so that the case is met by a remedy well matched with regard to its kind of action (first precaution), its necessary preparation and quantity (second precaution), and the proper time of its repetition (third precaution). Finally, when the physician knows in each the obstacles in the way of recovery and how to remove them, he is prepared to act thoroughly and to the purpose as a true master of the art of healing."

Here are the three points: First, the proper remedy; second, the proper dose, and third, the proper repetition. These points are the red thread which runs all through both the *Organon* and the *Chronic Diseases*. The whole *Organon* is taken up with teaching how to carry out what is portrayed in this third section. From section 3 to 71 Hahnemann teaches the nature of disease and the nature of the curative. The rest of the *Organon* discusses three questions, which are in section 71, viz.:

"1. How does the physician gain the knowledge of disease necessary for the purpose of cure?

"2. How does he gain his knowledge of the morbific power of drugs, as the implements designed for the cure of natural diseases?

"3. How does he apply these artificial morbid potencies most effectively in the cure of disease?"

From section 71 to 105 Hahnemann answers the first question. From section 105 to 146, he answers the second. From section 146 to the end he answers the third.

These same points Hahnemann displays even more clearly in section 246: "First, by careful selection of the most appropriate homœopathic medicine; secondly, by administering the medicine in the finest dose capable of restoring the vital force to harmonious activity without causing violent reaction; and thirdly, by repeating the finest dose of an accurately selected medicine at proper intervals."

In discussing his first precaution Hahnemann calls attention (on page 152 of *Chronic Diseases*) to the necessity "to inquire into the whole condition of the patient, the cause of the disease as the patient remembers it, his mode of life, the nature of his mind, the tone and character of his sentiments, his physical constitution, and especially the symptoms of the disease. This inquiry is made according to the rules laid down in the *Organon*. This being done, the physician then tries to discover the true homœopathic remedy."

Dunham, in his matchless language, compares the art of medicine with other arts, and says that the first thing an artisan should do is to become familiar with his tools and how to use them. If he should undertake to polish veneering by hewing with a broadax, he would most assuredly destroy his work. This would show extreme ignorance, yet no greater than Hahnemann charges upon allopathic physicians by their improper use of drugs. On page 146 of *Chronic Diseases* he says: "If the pernicious effects of the old method of cure merely resulted in dynamic derangements of the organism, the organism would soon recover from those effects after the treatment should have been discontinued for a time, or else homœopathic remedies would be able to neutralize those bad effects. But these effects never disappear. It is probable that the sensible and irritable fiber is unnaturally affected by the large and frequently repeated doses of allopathic medicine, and that the vital principle protects itself against their destructive agency, either by modifying or changing the viscera of organic life in such a manner as will effectually shield them against the assaults of subversive remedial agents. It is for a purpose like this, for instance, that the vital powers cover the delicate skin of the hand with a horny surface, in order to protect that part of the body against the bad effects which it might otherwise suffer from the contact with corrosive or otherwise injurious substances. In the same way, if the delicate viscera of organic life are assailed by medicines not in homœopathic relation with the disease, these viscera are protected by the vital principle by having their sensibility and irritability diminished. Moreover, the more delicate fiber is found abnormally thickened or hardened; the stronger fiber, on the contrary, consumed, or even annihilated, as is shown by the adventitious, irregular, or degenerate formations or growths which post-mortem examinations exhibit to us, and which are then attributed to the malignant character of the primitive disease."

On page 151, Hahnemann gives his idea of a strong homœopathic dose: "If the original symptoms of the disease continue with the same intensity in the succeeding days as in the beginning, or if this intensity increases, this is a sure sign that although the remedy may be homœopathic, yet the magnitude of the dose makes the cure impossible. The remedial agent, by its powerful action, not only neutralizes its genuine homœopathic effects, but establishes, moreover, in the system a medicinal disease by the side of the natural disturbance which is even strengthened by the medicine."

According to the homœopathic idea, it is not the direct action of the drug which starts the process of cure, but the reaction of the organism or the vital principle against the drug which does it. An overdose of the curative overwhelms the vital principle or the reaction of the organism so as to prevent a cure, and may leave it so changed as to preclude any hope of relief. Dr. Bayard puts this idea very aptly: "This power of reaction depends not upon the strength of the dose, but upon the exactitude of the prescription. The larger the dose, the more deleterious its direct action. The smallest dose compatible with the stimulus to reaction is the best. The higher the potency the more speedy the reaction." There are two ways by which to approach the proof of power of the potencies. One is to accept Hahnemann's teaching and begin with the use of the potencies, and prove them in practice, and the other is to begin as Hahnemann and most others have done, with the crude drug, and work up to the potencies. With careful observers the tendency is to go up higher and higher. Hahnemann went slow, and at last, supposing that he had reached the limit, gave as the ultimatum the thirtieth potency. Ever since his time the ultimatum has been going higher and higher. From Hahnemann's day to the present time there has been a constant conflict in regard to the potencies. He comprehended the whole situation, and saw into the future. He wrote: "Nothing is lost by giving even smaller doses than those which I have indicated. *The doses can scarcely be too much reduced*, provided the effects of the remedy are not disturbed by improper food." Are there many physicians who have been in practice until their hair has become sprinkled with gray, who cannot appreciate the following paragraph from the preface to the first edition of *Chronic Diseases:* "What would they have risked, if they had first followed my indications and had employed small doses? The most which could have befallen them was that these doses would be of no avail. It was impossible that they should do any harm. But instead of exhibiting small doses, they employed, from a want of sense and of their own accord, large doses for homœopathic use, thus exposing the lives of their patients, and arriving at truth by that circuitous route which I had traveled upon before them with trembling hesitation, but the end of which I had just reached with success. Nevertheless, after having done much mischief and having squandered the best period of their lives, they were obliged, when they were really desirous of curing disease, to resort to the only true method, which I had demonstrated to them a long time ago."

The third precaution is the everlasting vexing question of the repetition of the dose. Hahnemann's directions are always explicit. In all his works he refers to the others. In section 205 of our edition of the *Organon* he says: "I have endeavored to demonstrate the internal treatment of these diseases, as far as it was possible for a single physician to do, after many years of thought, observation, and experience, in my book on chronic diseases, to which I herewith refer the reader." In this he refers to the first edition of *Chronic Diseases.* In the second edition of *Chronic Diseases* he refers to the *Organon.* Authority in regard to the date of the writing the second edition of *Chronic Diseases* is Bœnninghausen in an article published in the *American Homœopathic Review*, vol. V, page 200, and Dr. Lippe. The preface to the fifth edition of the *Organon* has Hahnemann's signature and date (1833) attached. The value of these dates is simply to settle as to the latest authority. The second edition of *Chronic Diseases* settles this. The following quotations are from both the *Organon* and the *Chronic Diseases*, and are conclusive as to what he taught with reference to the repetition of dose.

First, from the *Organon*. In sections 246 and 247 he sums up for both acute and chronic diseases. "A very fine dose of a well-selected homœopathic remedy, if uninterrupted in its action, will gradually accomplish all of the curative effect it is capable of producing in a period varying from forty to one hundred days. But it rarely is uninterrupted, and besides, the physician as well as the patient usually desires to accelerate the cure by reducing this period of time, if possible, by one-half, one-quarter, or less. Experience has proved in numerous instances that such a result may actually be obtained under the following three conditions: First, by careful selection of the most appropriate homœopathic medicine (first precaution); secondly, by administering the medicine in the finest dose capable of restoring the vital force to harmonious activity without causing violent reaction (second precaution); and thirdly, by repeating the finest dose of an accurately selected medicine at proper intervals, such as are proved by experience to be most conducive to a speedy cure, and timed so as to prevent an injurious and revulsive counteraction of the vital force, whose action is to be tempered and modified in accordance with the morbific power of the medicine which is similar in effect to the natural disease (third precaution). Under these conditions the finest doses of the most nicely selected homœopathic medicines may be repeated with excellent and often astonishing effect at intervals of fourteen, twelve, ten, eight, and seven days. In chronic diseases assuming an acute form and demanding greater haste these spaces of time may be abbreviated still more, but in acute diseases the remedies may be repeated at much shorter intervals, for instance, twenty-four, twelve, eight, or four hours; and in the most acute diseases at intervals varying from one hour to five minutes. These periods are always to be determined by the more or less acute course of the disease, and by the nature of the remedy employed, in accordance with the more definite directions given in the explanatory note to the preceding paragraph."

Later in *Chronic Diseases*, page 154: "It takes forty and even fifty days before the medicine has completed its action." * * "The surest and safest way of hastening the cure is to let the medicine act as long as *the improvement of the patient continues*, were it even far beyond the period which is set down as the probable period of the duration of that action." In illustration of this he gives in a foot-note a case cured by Sepia. "On one occasion I gave Sepia against a chronic headache which came on at intervals. The attacks became both less frequent and less violent. Another dose stopped the headache for the period of one hundred days, from which I infer that the remedy acted during that time. At the end of one hundred days another slight attack came on. A third dose of Sepia was given, and it is now seven years since the headache has completely disappeared."

"*The fundamental rule in treating chronic diseases* is this: *To let the carefully selected homœopathic antipsoric act as long as it is capable of exercising a curative influence and there is a visible improvement going on in the system.* This rule is opposed to the hasty prescription of a new, or *the immediate repetition of the same remedy.*" On page 160 he is more definite: "A second dose of the remedy may be given *immediately after* the first, when the remedy had been chosen with strict regard to its homœopathic character and had produced a good effect, but had not acted long enough to cure the disease. This occurs but seldom in chronic diseases; but it frequently occurs in acute diseases and in those chronic diseases that border upon the acute. The same remedy may be given a second time *when the improvement which the first dose had produced by causing the morbid symptoms gradually to become less frequent and less intense ceases to continue after the lapse of fourteen, ten, or seven days; when it becomes, therefore, evident that the medicine has ceased to act, the condition of the mind is the same as before, and no new or troublesome symptoms have made their appearance. All this would show that the same remedy is again indicated.*"

The importance with which Hahnemann regarded these directions is shown in all his writings. He repeats them everywhere. A fit closing to this paper will be some extracts from the *Organon*, concluded with a single one from *Chronic Diseases.*

*Organon*, section 245: "Perceptible and continued progress of improvement in acute or chronic disease is a condition which, as long as it lasts, invariably counter-indicates the repetition of any medicine whatever, because the beneficial effect which the medicine continues to exert is rapidly approaching its perfection. Under these circumstances every new dose of any medicine, even if the last one that proved beneficial, would disturb the process of recovery."

248: "The dose of the same medicine is to be repeated several times if necessary, but only until recovery ensues, or *until the remedy ceases to produce improvement; at that period the remainder of the disease, having suffered a change in its group of symptoms, requires another homœopathic medicine.*"

Immediately following this direction should come this from page 156 of *Chronic Diseases*, and this should be printed on the outer and inner walls; in the pocket-book and the medicine-case; in every place where the eye can rest: "The whole cure fails if the antipsoric remedies, which have been prescribed for the patient, are not permitted to act uninterruptedly to the end. Even if the second antipsoric should have been selected with the greatest care, it cannot replace the loss which the rash haste of the physician has inflicted on the patient. The benign action of the former remedy, which was about manifesting its most beautiful and most surprising results, is probably lost to the patient forever."

